# Unique ER PR expression pattern in breast cancers with *CHEK2* mutation: a hormone receptor and HER2 analysis based on germline cancer predisposition genes

**DOI:** 10.1186/s13058-022-01507-1

**Published:** 2022-02-08

**Authors:** Grace Wei, Mingxiang Teng, Marilin Rosa, Xia Wang

**Affiliations:** 1 University of South Florida Morsani College of Medicine, Tampa, FL USA; 2 Department of Biostatistics and Bioinformatics, H. Lee Moffitt Cancer Center and Research Institute, Tampa, FL USA; 3 Department of Pathology, H. Lee Moffitt Cancer Center and Research Institute, Tampa, FL USA; 4 GeneHome Hereditary Cancer Screening Clinic, H. Lee Moffitt Cancer Center & Research Institute, 10920 N. McKinley Drive, Tampa, FL 33612 USA

**Keywords:** Breast cancer, Germline cancer predisposition gene, ER, PR, HER2, Hormone receptor expression, Tumorigenesis

## Abstract

**Purpose:**

Estrogen-receptor (ER) and progesterone-receptor (PR) expression levels in breast cancer, which have been principally compared via binomial descriptors, can vary widely across tumors. We sought to characterize ER and PR expression levels using semi-quantitative analyses of receptor staining in germline pathogenic variant (PV) carriers of cancer predisposition genes.

**Methods:**

We conducted a retrospective chart review of patients who underwent germline genetic testing for cancer predisposition genes at a tertiary cancer center genetics clinic. We performed comparisons of semi-quantitative ER and PR percentage staining levels across carriers and non-carriers of cancer predisposition genes.

**Results:**

Breast cancers from *BRCA1* PV carriers expressed significantly lower ER (15.2% vs 78.2%, *p* < 0.001) and lower PR (6.8% vs 41.1%, *p* < 0.001) staining compared to non-PV carriers. Similarly, breast cancers of *BRCA2* (66.7% vs 78.2%, *p* = 0.005) and *TP53* (50.6% vs 78.2%, *p* = 0.015) PV tumors also displayed moderate decreases in ER staining. Conversely, *CHEK2* tumors displayed higher ER (93.1% vs 78.2%, *p* = 0.005) and PR (72% vs 48.8%, *p* = 0.001) staining when compared to non-PV carriers. We observed a wide range of dispersion across the ER and PR staining levels of the carriers and noncarriers. ER and PR ranges of dispersion of *CHEK2* tumors were uniquely narrower than all other groups.

**Conclusion:**

The findings of our study suggest that precise expression levels of ER and PR in breast cancers can vary widely. These differences are further augmented when comparing expression staining across PV and non-PV carriers, suggesting potentially unique tumorigenesis and progression pathways influenced by germline cancer predisposition genes.

## Introduction

Estrogen, progesterone, and HER2 receptor statuses are important factors influencing breast cancer progression and have served as cornerstones for breast cancer classification and treatment. In recent decades, genomic instability has risen as a predictor of cancer outcomes and offers opportunity for targeted treatment. In a large cohort of breast cancer patients, genome instability profiles were found to predict mortality outcomes independent of clinicopathological parameters [1]. A number of breast cancer predisposition genes (*ATM, BRCA1, BRCA2, CHEK2, TP53,* and *PALB2*) are important gatekeepers to maintaining DNA replication fidelity by regulating DNA damage repair. Germline pathogenic variants (PVs) in these genes significantly increase the lifetime risk of breast cancer and the average age of cancer onset in these PV carriers is often earlier than that in the general population.

ER and PR expression in breast cancer have primarily been studied via binomial comparisons, ER-positive (ER +) and ER-negative (ER-). Because estrogen is a key regulator of progesterone receptor synthesis in mammary tissue, the exact expression level of PR is often overlooked. However, PR levels can be quite variable even when ER is strongly expressed in breast tumor cells and the expression levels for both ER and PR can vary greatly in a tumor designated “ER/PR positive.” It is possible that differences in levels of ER and PR expression may lead to differential treatment responses and survival outcomes.

Precise characterization of the ER and PR hormone receptors and HER2 expression levels of breast cancer arising in germline genetic PV carriers can help improve our prognostic understanding of these tumors and shed light on the tumorigenesis process. To address this gap, we investigated the ER and PR expression levels using semi-quantitative data analysis of ER and PR staining percentages in germline PV carriers of moderate and high penetrant breast cancer genes. To our knowledge, a semi-quantitative analysis of the hormonal receptor expression levels in this patient population has not yet been previously reported in the literature.

## Methods

We conducted a retrospective chart review of a cohort (cohort A) composed of consecutive cases of 684 breast cancer patients who underwent germline panel genetic testing for cancer predisposition genes at the genetics clinic in Moffitt Cancer Center (MCC). Testing occurred between January 2015 and February 2018. We also reviewed another cohort (cohort B) composed of consecutive cases of individuals who had a prior diagnosis of breast cancer and pursued genetic high-risk cancer screening and surveillance in the MCC GeneHome clinic between March 2017 and September 2020 after being found to carry germline cancer predisposition PVs. These cases were combined with PV carriers in cohort A to form the study group. Cancer predisposition genes studied included moderate and high penetrant breast cancer genes, including *BRCA1, BRCA2, PALB2*, *TP53*, *PTEN*, *CDH1*, *ATM*, and *CHEK2*. Because mismatch repair genes are often tested in this patient population, carriers with Lynch syndrome mismatch repair gene PVs, namely *MLH1, MSH2, MSH6* and *PMS2*, were included in this study. Individuals who tested negative for known cancer predisposition genes served as the reference (control) group for the analysis. We analyzed potential effects from multiple clinical variables, such as age, race, etc., and observed no significant bias or batch effects from these variables comparing the two cohorts.

Combining cohort A and cohort B, we selected candidate cases for the study group based on the following inclusion criteria: adult female patients (≥ 18 years old) with a (1) clinical breast cancer diagnosis, (2) cancer predisposition germline genetic test result, (3) available records and data for primary breast cancer, and 4) available clinical grade ER and PR data expressed by the percentage of staining, as well as available HER2 status expressed as binomial category, positive or negative, determined by immunohistochemistry (Ventana PATHWAY® system) or by in situ hybridization (FISH or DISH). To minimize confounding, cases with PV on greater than one breast cancer risk gene were excluded from the analysis. Individual cases with PVs on both breast cancer genes and MMR genes were excluded. Duplicating cases found in both cohort A and cohort B were also excluded.

ER and PR staining levels, based on the percentage of cell staining, were compared between PV carriers and non-PV carriers via Mann Whitney U at *p* > 0.05. Variable staining percentages were studied using coefficient of variation to characterize the degree of dispersion for each study group. Binomial ER, PR and HER2 expression (positive and negative) comparisons were assessed via chi-square analysis. This study was approved by the Moffitt Cancer Center IRB.

## Results

Our cohort included a total of 613 cases. The average age at clinic presentation of non-PV carriers (n = 364) was 55.3 years and the average age at breast cancer diagnosis was 52.7 years. Among germline PV carriers (n = 249), the average at clinic presentation was 50.5 years and average age at cancer diagnosis was 47.1 years (Table 1). When compared with non-PV carriers, breast tumors from *BRCA1* PV carriers expressed significantly lower ER (15.2% vs 78.2%, *p* < 0.001) and lower PR (6.8% vs 41.1%, *p* < 0.001) staining. In addition, *BRCA2* (66.7% vs 78.2%, *p* = 0.005) and *TP53* (50.6% vs 78.2%, *p* = 0.015) PV tumors also displayed moderately lower ER staining. Contrarily, *CHEK2* tumors displayed higher ER (93.1% vs 78.2%, *p* = 0.005) and PR (72% vs 48.8%, *p* = 0.001) staining when compared to the reference group (Table 2). Furthermore, HER2-negative breast cancers were significantly more prevalent among *BRCA1* (98.2% vs 77.7%, *p* < 0.001) and *BRCA2* (95.0% vs 77.7%, *p* = 0.001) PV carriers than non-PV carriers (Table 3).

**Table 1:** Age at presentation and breast cancer diagnosis by germline PV carrier status

	Age at presentation average (SD)(Median)	Age at cancer diagnosis average (SD)(Median)
NonPV carriers	55.3 (± 11.8) (55)	52.7 (± 21.4) (49)
PV carriers	50.5 (± 13.1) (50)	47.1 (± 12.1) (46)

**Table 2:** Breast cancer ER/PR staining percentage based on germline PV status

Germline PV	Case no. (N)	ER%					PR%				
		Mean	SD	*p* value	CV	CV *p* value	Mean	SD	*p* value	CV	CV *p* value
BRCA1	57	15.2	33.6	0.000	2.21	0.000	6.8	20.9	0.000	3.05	0.000
BRCA2	60	66.7	38.9	0.000	0.58	0.007	44.7	38.0	0.161	0.85	0.934
PALB2	22	68.2	39.5	0.111	0.58	0.087	39.5	34.4	0.156	0.87	0.867
ATM	22	83.2	29.2	0.540	0.35	0.306	55.8	39.3	0.344	0.70	0.539
TP53	21	50.6	47.5	0.015	0.94	0.000	40.6	45.0	0.397	1.11	0.235
CHEK2	39	93.1	17.8	0.005	0.19	0.000	71.7	35.4	0.001	0.49	0.019
MMR	19	60.9	47.7	0.281	0.78	0.000	40.4	42.5	0.445	1.05	0.324
PTEN	6	98.0	3.82	NC	NC	NC	87.0	17.85	NC	NC	NC
CDH1	3	80.0	52.9	NC	NC	NC	70.0	40.4	NC	NC	NC
Control/reference		78.2	34.4		0.44		41.1			0.84	

PV: Pathogenic variant; SD: Standard deviation; CV: Coefficient of variation; MMR: Mismatch repair genes

**Table 3:** Breast cancer HER2 staining percentage by germline PV status

	Case no. (N)	HER2−	HER2 +	*p* value
BRCA1	56	55 (98.2%)	1 (1.8%)	0.000
BRCA2	60	57 (95.0%)	3 (5.0%)	0.001
PALB2	22	20 (90.9%)	2 (9.1%)	0.186
ATM	22	17 (77.3%)	5 (22.7%)	1.000
TP53	21	13 (61.9%)	8 (38.1%)	0.113
CHEK2	39	31 (79.5%)	8 (20.5%)	1.000
MMRs	18	11 (61.1%)	7 (38.9%)	0.146
PTEN	6	6 (100%)	0 (0.0%)	0.346
CDH1	3	3 (100%)	0 (0.0%)	1.000
Control/Reference	355	276 (77.7%)	79 (22.3%)	–

**Fig. 1:**
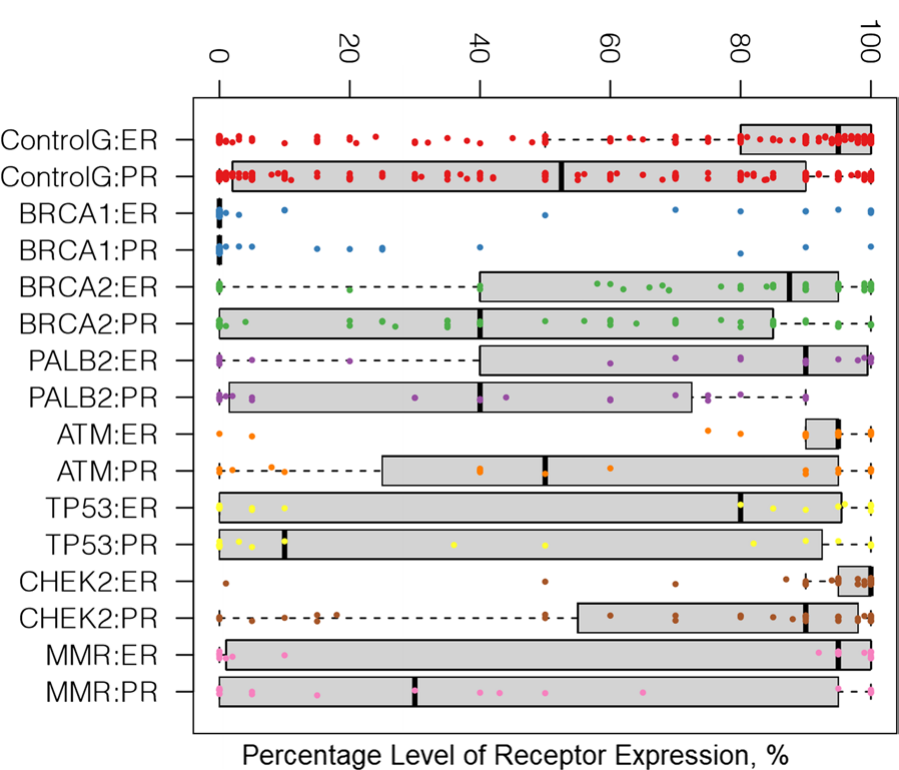
Breast tumor ER/PR staining percentages by germline genes

These results demonstrated significant dispersion in ER and PR percentage levels among both PV and non-PV carrier groups. However, the dispersion pattern of ER and PR percentage levels yielded particularly unique features for certain PV carriers (Table 2) (Fig. 1). Most strikingly, *CHEK2* PV tumors exhibited significantly more staining and significantly lower dispersion of ER and PR values among all PV carriers or when compared with the reference group. It was evident that the lower standard deviation and lower coefficient of variance (CV) were considerably different than most other groups (ER: mean = 93.1 SD = 17.8 *p* = 0.005, CV = 0.19, *p* < 0.0001; PR: mean = 71.7 SD = 35.4 *p* = 0.001, CV = 0.49, *p* = 0.019).

HER2 positive (HER2 +) tumors were less common in *BRCA1* and *BRCA2* tumors (Table 3). TNBCs (triple negative breast cancers, ER-, PR-, and HER2-) were more commonly found in carriers with *BRCA1*, *BRCA2* or *PALB2* PVs (Table 4). ER + /PR- tumors appeared to be more common in non-PV tumors, however, differences did not reach statistical significance. Further, very few ER-/PR + tumors in PV or non-PV tumors were observed. Among these ER-/PR + tumors, the levels of PR staining were very low (< 5%). Regarding overall cancer history, prevalence of ER + /PR- breast cancers were significantly greater in patients with additional cancer diagnoses (17.5%) compared to patients with solely breast cancer diagnoses (10.6%) (*p* = 0.02). These additional cancer diagnoses included head and neck, thyroid, melanoma and non-melanoma skin cancers, pancreatic, renal, bladder, prostate, esophageal, uterine, ovarian, cervical, neuroendocrine, lymphoma, multiple myeloma, and sarcoma.

**Table 4:** TNBC, ER + /PR−, ER−PR + , based on germline PV

Germline PV	Case No	TNBC	ER + /PR−	ER-/PR +	*p* value (TNBC)
BRCA1	57	40 (70.2%)	5 (8.8%)	2 (3.5%)	0.000
BRCA2	60	12 (20.2%)	5 (8.3%)	0 (0%)	0.000
PALB2	22	3 (13.6%)	1 (4.5%)	0 (0%)	0.003
ATM	22	1 (4.5%)	2 (9.1%)	0 (0%)	0.210
TP53	21	0 (0%)	2 (9.5%)	1 (4.8%)	1.000
CHEK2	39	0 (0%)	6 (15.4%)	0 (0%)	0.415
MMR	19	1 (5.3%)	2 (10.5%)	0 (0%)	0.185
PTEN	6	0 (0%)	1 (16.7%)	0 (0%)	1.000
CDH1	3	1 (33.3%)	0 (0%)	0 (0%)	1.000
Control	364	3 (0.8%)	82 (22.5%)	13 (3.6%)	

## Discussion

Estrogen receptors and progesterone receptors are overexpressed in the majority of human breast cancers. Blocking estrogen facilitated cell proliferation has been a major component of the breast cancer treatment for more than three decades. Approximately 82% of breast cancers are ER + and/or PR + (HR positive means ER + and/or PR + , TNBC 12.2%; HR−/HER2 + 4.5%, TPBC 10.3%, HR + /HER2− 72.2%) [2]. Estrogen is a known key regulator of progesterone receptor synthesis in mammary tissue, thus anti-hormone breast cancer treatment has generally been guided by ER level, irrespective of PR level. However, the expression levels of ER and PR can vary significantly and even become divergent in some breast tumors, such as those with ER + /PR− or ER-/PR + statuses. Thus, there is a growing suspicion that the effects of estrogen, progesterone and their receptors in breast tissue are not equal. Indeed, multiple reports have suggested poor prognosis of ER + /PR− breast cancer in older affected populations [3].

There is growing knowledge that hormone receptor expression levels are influenced by germline breast cancer predisposition gene defects. It is known that carriers of *BRCA1* PVs tend to develop ER-/PR-/HER2-, i.e. triple negative breast cancers (TNBC) [4], while carriers of *BRCA2* PVs tend to develop ER + /PR + breast cancers, similar to sporadic forms lacking germline predispositions. TNBCs are clinically aggressive and occur more often in younger women. *BRCA2* defective tumors are more consistent with luminal B molecular subtype, a subtype generally only found in one-fifth of non-selected breast tumors. The prognosis of luminal B type is also inferior to the luminal A type found in more than 50% of breast cancers [5, 6].

The roles of hormone and hormone receptor expression during tumorigenesis and progression are complex [7, 8]. Population studies have suggested a difference in estrogen and progesterone influences on breast cancer disease processes. Chlebowski et al. demonstrated dramatic differences on the impact of estrogen plus progesterone (E + P) therapy compared to estrogen alone on breast cancer incidence, whereby estrogen and progesterone combined increased breast cancer risk and estrogen alone decreased the risk [9].

The results of ER and PR expression patterns revealed by our study, particularly the higher degree of expression and decreased dispersion of ER/PR in *CHEK2* tumors, suggests unique mechanisms of tumorigenesis, such as reduced diversity in tumor evolution on the backdrop of the CHEK2 haplo-insufficiency. Many tumor suppressor genes, such as *ATM*, *CHEK2*, *BRCA1*, *BRCA2*, *PALB2*, and *TP53* function in a vast network of DNA damage repair responses and cell cycle check point regulations. Notably, *CHEK2* functions in the same DNA repair landscape as *BRCA1*, *BRCA2*, *PALB2*, and *TP53*, however, the ER and PR expression levels of *CHEK2* tumor differ significantly from all other tumors, including PV and non-PV tumors.

Clinically, the variability of ER/PR expression levels may be tied to responsiveness to anti-hormonal therapies. The higher levels of dispersion of ER/PR expression may indicate more dynamic tumor evolution, rendering tumors to be more or less responsive to certain systemic or radiation therapies. Future research utilizing multi-institutional large cohort and longitudinal studies are needed to better elucidate differences in responsiveness to therapy and survival between various germline PV carriers. This improved understanding of the mechanisms underlying ER/PR expression levels and the associated dispersion of levels may benefit cancer precision care, diagnostics, surveillance or treatment for women at risk for breast cancer.

The significance of the results of this study should be viewed in light of study limitations. Instead of an unselected sporadic breast cancer population, the reference (control) group represents a unique population of patients who developed breast cancer at a relatively younger age and frequently with a family history of breast cancer. The reference cohort was mainly composed of females who came to pursue cancer genetic counseling and testing. Suspicions for hereditary risk of breast cancer are often triggered by personal and/or family history of early age at breast cancer diagnosis, multiple relatives affected with breast cancer, or other cancers associated with genetic predisposition, such as ovarian cancer, endometrial cancer, prostate cancer or pancreatic cancer. One of the major criteria to suspect *BRCA1* germline pathogenic variants and recommend genetic test is TNBC, even if it was diagnosed at an older age. This likely is responsible for the observation of more ER-/PR- cases reported (24.4%) in this cohort than average non-selected breast cancers in the general population (16.8%). Potential for sample selection bias (potential batch differences) between cohort A and B were minimized, as all participants were drawn from the same population seeking cancer predisposition genetic testing.

This study confirmed previous known patterns, including that ER-/PR- breast cancers are more often seen in *BRCA1* PV carriers, in younger females, whereas ER + /PR + breast cancers are more often seen in *BRCA2* PV carriers [^10^]. ER + /PR + tumor occur more commonly in other high and moderate breast risk gene PV carriers. Germline defective *BRCA1* is a known strong driver for the ER-/PR- breast cancer, and approximately 80% are ER−/PR− in women carrying *BRCA1* PVs. *BRCA1* PV is also strongly associated with a very early onset breast cancer under age 35. Finally, ER + /PR- breast cancers were found in 10.6% of cases with one breast cancer only compared to 17.5% with breast cancer and an additional cancer diagnosis, *p* = 0.02.

## Conclusion

Among breast cancer developed in PV and non-PV carriers, ER and PR staining levels demonstrated very wide ranges of dispersion. The ER ranges of dispersion were significantly wider for *BRCA2*, *TP53*, and MMR genes tumors compared to the reference group, while ER and PR ranges of dispersion of *CHEK2* tumors were significantly narrower than all other groups including the non-PV tumor reference groups. Some of the tumor suppressor PV carriers (*BRCA1*, *BRCA2*, *TP53*) developed tumors with less ER expression, while *CHEK2* tumors exhibited higher expression for both ER and PR. The results of this study suggest unique tumorigenesis pathways in *CHEK2* germline PV carriers. In addition, HER2-positive tumors were significantly less common in *BRCA1* and *BRCA2* tumors when compared with non-PV carriers.

## Data Availability

The datasets used and/or analysed during the current study are available from the corresponding author on reasonable request.
